# Mercury-induced hepatotoxicity in zebrafish: *in vivo *mechanistic insights from transcriptome analysis, phenotype anchoring and targeted gene expression validation

**DOI:** 10.1186/1471-2164-11-212

**Published:** 2010-03-30

**Authors:** Choong Yong Ung, Siew Hong Lam, Mya Myintzu Hlaing, Cecilia Lanny Winata, Svetlana Korzh, Sinnakaruppan Mathavan, Zhiyuan Gong

**Affiliations:** 1 Department of Biological Sciences, 14 Science Drive 4, National University of Singapore, 117543 Singapore; 2 Genome Institute of Singapore, Agency for Science Technology and Research, Genome, 60 Biopolis Street, 138672 Singapore

## Abstract

**Background:**

Mercury is a prominent environmental contaminant that causes detrimental effects to human health. Although the liver has been known to be a main target organ, there is limited information on *in vivo *molecular mechanism of mercury-induced toxicity in the liver. By using transcriptome analysis, phenotypic anchoring and validation of targeted gene expression in zebrafish, mercury-induced hepatotoxicity was investigated and a number of perturbed cellular processes were identified and compared with those captured in the *in vitro *human cell line studies.

**Results:**

Hepato-transcriptome analysis of mercury-exposed zebrafish revealed that the earliest deregulated genes were associated with electron transport chain, mitochondrial fatty acid beta-oxidation, nuclear receptor signaling and apoptotic pathway, followed by complement system and proteasome pathway, and thereafter DNA damage, hypoxia, Wnt signaling, fatty acid synthesis, gluconeogenesis, cell cycle and motility. Comparative meta-analysis of microarray data between zebrafish liver and human HepG2 cells exposed to mercury identified some common toxicological effects of mercury-induced hepatotoxicity in both models. Histological analyses of liver from mercury-exposed fish revealed morphological changes of liver parenchyma, decreased nucleated cell count, increased lipid vesicles, glycogen and apoptotic bodies, thus providing phenotypic evidence for anchoring of the transcriptome analysis. Validation of targeted gene expression confirmed deregulated gene-pathways from enrichment analysis. Some of these genes responding to low concentrations of mercury may serve as toxicogenomic-based markers for detection and health risk assessment of environmental mercury contaminations.

**Conclusion:**

Mercury-induced hepatotoxicity was triggered by oxidative stresses, intrinsic apoptotic pathway, deregulation of nuclear receptor and kinase activities including Gsk3 that deregulates Wnt signaling pathway, gluconeogenesis, and adipogenesis, leading to mitochondrial dysfunction, endocrine disruption and metabolic disorders. This study provides important mechanistic insights into mercury-induced liver toxicity in a whole-animal physiology context, which will help in understanding the syndromes caused by mercury poisoning. The molecular conservation of mercury-induced hepatotoxicity between zebrafish and human cell line reveals the feasibility of using zebrafish to model molecular toxicity in human for toxicant risk assessments.

## Background

Mercury is a highly hazardous pollutant with an estimated global natural mercury emission of 1,800-5,800 tons per annum [1] and global anthropogenic mercury emission to the atmosphere was estimated to be 2,190 tons in 2000 [2]. Since mercury is ubiquitous in the environment, it is nearly impossible for most humans and animals to avoid exposure to some forms of mercury, be it elementary, organic or inorganic. Mercury, through biotransformation and bioaccumulation, has found its way through the food chain to humans. Anthropogenic activities and industrialization are also sources of mercury pollution that had resulted in several catastrophe of mercury poisoning in Japan [3], the Amazon basin [4] and Iraq [5], and recently many high-risk sites have been identified in Asia [1]. Moreover, mercury pollution and poisoning have imposed a huge economic cost on environmental remediation and public health [6,7].

All forms of mercury cause toxic effects in a number of tissues and organs depending on the chemical form of mercury as well as the level, duration and the route of exposure [8-11]. Exposure to mercury compounds typically occurs by inhalation or ingestion. Ingested mercury is absorbed in the gastrointestinal tract and it is distributed to all tissues in about 30 h while inhaled through mercury vapor accumulates in red blood cells and is carried to all tissues in the body in less than 24 h [11]. Mercury undergoes extensive biliary-hepatic cycling [12]. It is secreted into bile and partly reabsorbed into the portal circulation and thereby returned to the liver. The high mobility of mercury in the body is attributed to the formation of water-soluble mercury complexes that are mainly, if not exclusively, attached to the sulfur atom of thiol groups such as glutathione [11]. The glutathione moiety is degraded in the bile duct and gall bladder to a dipeptide and finally to an L-cysteine mercury complex before entering the circulatory system [12]. It is in the form of the L-cysteine complex that mercury enters the endothelial cells of the blood-brain and placental barriers. Mercury can gradually accumulate in the central nervous system and kidney, thus causing damage to these organs. Hence, there have been several molecular mechanistic studies on neurological [13] and renal [14] toxicities induced by mercury. However, despite of the fact that the extensive biliary-hepatic cycling of mercury [12] and of some evidence suggesting that liver plays a role in renal tubular uptake of mercury [14], little is known with regard to the mechanism of mercury-induced hepatotoxicity. Based on the role of liver in mercury biotransforming and cycling, as well as its central roles in the control and synthesis of critical blood constituents that affect whole body physiology, we hypothesized that mercury exposure can cause liver injury that may lead to other syndromes. In view of the prevalence of mercury exposure in humans, understanding of the mechanism of mercury-induced hepatotoxicity is therefore important for elucidating its impact on liver health.

The advent of omics technologies such as genomics and transcriptomics has enabled the simultaneous assessment of expression profiles of thousands of genes that respond to a toxic compound within a particular cell type, tissue, or an organism [15]. The zebrafish is an increasingly popular model not only for vertebrate development [16] but also for understanding human diseases [17] and toxicology [18]. Many physiological and molecular similarities in xenobiotic metabolism and adaptive response to toxicant insults have been found between zebrafish and mammals, making the zebrafish an ideal toxicology model in toxicology [19,20]. Although zebrafish has become popular as an *in vivo *system to model human diseases and toxicities, toxicogenomic researches using zebrafish seem not to have been extensively conducted. We and others have shown that zebrafish responded biologically to chemicals, such as small molecules, drugs and environmental toxicants, in a similar manner as mammals [21-24]. The availability of zebrafish in large numbers, its small size and easy husbandry makes zebrafish a cost-effective model when compared to the rodent model for toxicological studies. In addition, the amenability of the zebrafish system to various molecular techniques and the vast genomic resources, including the near-completed zebrafish genome project and available zebrafish microarrays, make it a highly versatile system for toxicogenomic studies. Moreover, fish have long been used as sentinels for biomonitoring of aquatic environmental pollutants and are good indicators of mercury pollution [19,20].

To our knowledge, there is no research report to elucidate the *in vivo *hepatotoxicity mechanism induced by mercury as well as its induced syndromes. Recent work by Gonzalez *et al *had indicated that the liver of the zebrafish is one of the main organs of mercury accumulation during the first 7 days of exposure to methylmercury [25]. In this study, we aimed to investigate the *in vivo *mechanism of mercury-induced hepatotoxicity using the zebrafish as a model and compared it with other published data. To date, most mechanistic studies of mercury-induced toxicity in human/mammals have been performed either *in vitro *or *in vivo *using non-liver tissue. However, mechanistic insight obtained from the *in vitro *systems can be quite different from those obtained from the *in vivo *environment because of the lack of coordination of biological processes in whole organism. As the liver is the main organ that performs detoxification processes as well as regulation of metabolic pathways, it is important to understand toxicity effects and malfunctions that may implicate metabolic disorders induced by some toxicants in liver *in vivo*.

Here, using the DNA microarray technology, we captured the kinetics of hepatic transcriptome changes in zebrafish upon mercury exposure in order to generate a global transcriptome view of mercury-induced hepatotoxicity. Using gene set enrichment analysis (GSEA) and knowledge-based data mining, we compared our findings with those reported in human liver cell line and/or in mammalian model. We performed phenotype anchoring by linking histological analyses to the transcriptome data and subsequently performed gene expression validation using real-time PCR on a separate set of samples from seperate mercury exposure experiments. Taken together, we proposed a plausible *in vivo *mechanistic model of mercury-induced hepatotoxicity that could lead to syndromes related to mitochondrial dysfunction, endocrine disruption and metabolic disorders.

## Results and discussion

### Characterization of mercury-induced hepatotoxicity responses in zebrafish from transcriptome analysis

Preliminary acute toxicity test was first performed to determine the appropriate concentrations of mercuric chloride (HgCl_2_) for DNA microarray experiments. Fishes were treated with different concentrations of HgCl_2 _ranging from 100 to 600 μg/L for 7 days. Acute toxicity and motility test for HgCl_2 _exposure treatment revealed that 600 μg/L HgCl_2 _caused all the fish to die within 2 days, while treatment at concentrations of 200 μg/L produced 90% of survival rate up to 7 days (See Additional file 1). All surviving fish treated with 200 μg/L displayed normal swimming behavior without obvious adverse effect. Therefore, 200 μg/L of HgCl_2 _was chosen for the subsequent DNA microarray experiments as the concentration was sufficient to induce acute toxicity with minimal mortality.

Bioaccumulation of HgCl_2 _was not determined in the present study. In a similar study recently published [26], 0.15 mg/L HgCl_2 _was used for exposure in another tropical freshwater fish, *Brycon amazonicus*, and 10.46 mg Hg per kg liver from treated fish was detected in fish liver at 96 h and thus a bioaccumulation factor of about 100 in the liver was estimated.

Based on the transcriptome data, genes that were significantly regulated by mercury were identified at different time points (8 h, 24 h, 48 h and 96 h) with p-value < 0.05 and mapped to human homologous genes (See Additional file 2). Compared to 8 h time point, the number of genes significantly deregulated (both up- and down-regulated) at 24 h increased dramatically and a further increase was observed at 96 h time point. The increasing numbers of differentially expressed genes may serves as a rough indication that more biological processes were deregulated with the increase of duration of mercury exposure up to 96 h.

Gene set enrichment analyses (GSEA), which employs a statistical algorithm to compare the entire ranked list of genes through transcriptome profiles with defined knowledge-based gene sets obtained from established biological knowledge [27, see Methods], was used to determine biological pathways perturbed by HgCl_2_. Biological pathways that are statistically enriched (nominal p-value [NP] <0.1 or false discovery rate [FDR] <0.3) from whole transcriptome analyses at the four time points (8 h, 24 h, 48 h and 96 h) of HgCl_2 _treatment using GSEA are shown in Table 1. The activities of the enriched pathways are indicated in normalized enrichment scores (NES) as described in the Methods section. Positive and negative NES values indicate up- and down-regulation of genes associated with pathways, respectively. In addition, we also explored canonical pathways that are enriched using the Ingenuity Pathway Analysis (IPA) System http://www.ingenuity.com/. We considered early (8 and 24 h) and late (48 and 96 h) stages in this analysis and the top 10 enriched canonical pathways were compared with results from GSEA (data not shown). Results from both GSEA and IPA suggest that pathways associated with protein degradation, stress, acute phase responses, mitochondrial dysfunction, and cell damage are enriched (data not shown).

**Table 1:** Gene Set Enrichment Analysis (GSEA) of liver transcriptome of HgCl_2_-treated zebrafish.

	Up-regulated	NES	Down-regulated	NES
				
				
**8 h**			Electron transport chain	-2.1201 (15)
			Mitochondrial fatty acid beta-oxidation	-1.4134 (10)
			Nuclear receptor signaling pathway	-1.484 (7)
			Extrinsic apoptotic pathway	-1.5717 (4)
				
**24 h**	Proteasome pathway	1.7531 (7)	Mitochondrial fatty acid beta-oxidation	-1.7489 (10)
	Complement activation (classical)	1.6751 (2)		
				
**48 h**	Proteasome pathway	2.0369 (8)	Mitochondrial fatty acid beta-oxidation	-1.5011 (6)
	Electron transport chain	1.6143 (15)	Nuclear receptor signaling pathway	-1.689 (8)
				
**96 h**	Proteasome pathway	1.9855 (5)	Non-substrate GSK3 interacting proteins	-1.533 (5)
	Complement activation (classical)	1.435 (2)	Cell cycle	-1.3784 (12)
	DNA damage signaling pathway	1.4009 (14)	Wnt signaling pathway	-1.337 (8)
	Actin pathway	1.7501 (3)		
	Fatty acid synthesis	1.5715 (4)		
	Intrinsic apoptotic pathway	1.401 (4)		
	Hypoxia-induced factor pathway	1.3243 (5)		
	Cell motility	1.2936 (5)		
	Gluconeogenesis	1.0784 (7)		
	Electron transport chain	1.6832 (18)		

Biological pathways that are statistically enriched with false discovery rate (FDR) < 0.3 or nominal p-value (NP) < 0.1 are shown. Values of normalized enrichment score (NES) indicate the activities of enriched pathways with positive and negative NES shows up- and down-regulation, respectively. Number of leading edge genes for each pathway is given in bracket besides the NES values.

The transcriptome analysis using GSEA revealed that genes associated with electron transport chain, mitochondrial fatty acid beta-oxidation, nuclear receptor signaling and apoptotic pathway were down-regulated as early as 8 h of HgCl_2 _exposure. This was followed by up-regulation of genes associated with the complement system and proteasome pathway, suggesting increased activity of these processes at 24 h. Major processes that were affected throughout the latter three time points of HgCl_2 _treatments (24 h, 48 h and 96 h) were proteasome pathway, electron transport chain, and mitochondrial fatty acid beta-oxidation (Table 1). Interestingly, electron transport chain that was initially down-regulated at 8 h were subsequently up-regulated at 48 h and 96 h, implying an increased need of intracellular energy production presumably to compensate the early down-regulation and to meet the energy requirement of the adaptive response to HgCl_2 _exposure. At 96 h of HgCl_2 _exposure, more biological pathways were significantly affected such as up-regulation of actin pathway, fatty acid synthesis, DNA damage signaling pathway, classical complement activation, intrinsic apoptotic pathway, cell motility, hypoxia-induced factor pathway, and gluconeogenesis, as well as down-regulation of cell cycle, non-substrate Gsk3 interacting proteins, and Wnt signaling pathway.

Taken together, the transcriptome analysis revealed that early targets of mercury-induced hepatotoxicity involved mitochondrial processes (electron transport chain and fatty acid beta-oxidation), apoptotic pathway and nuclear receptor signaling. The following up-regulation of complement system and proteasome pathway suggest activation of acute phase response and increased degradation of damaged proteins within 24 h of HgCl_2 _exposure. By 96 h, several critical processes associated with cellular maintenance, stress, survival and metabolism were clearly deregulated in the liver. Earlier work by Gonzalez *et al *[25] using the zebrafish to elucidate effects of methylmercury on gene expression in liver, skeletal muscle, and brain indicated genes involved in mitochondrial metabolism, detoxification process, DNA repair and apoptosis were generally deregulated. Hence, there are similar observed toxicity effects between inorganic mercury and organic mercury such as methylmercury. Thus, it is important to further understand the mechanism of their *in vivo *toxicity in future works.

### Comparative analysis revealed some common mercury-induced processes in both zebrafish liver and human liver cell line

In order to determine molecular conservation of mercury-induced hepatotoxicity, we compared pathways that were induced or suppressed by HgCl_2 _between zebrafish and human cell line using GSEA. Transcriptome data of liver cell line (HepG2) exposed to 20 μM of HgCl_2 _for 6 h were obtained from the Gene Expression Omnibus (GEO Accession GSE6907, http://www.ncbi.nlm.nih.gov/geo/) and used for comparative study with our zebrafish transcriptome data. Deregulated processes with FDR< 0.3 or NP < 0.1 were selected. Comparative GSEA revealed that DNA damage signaling and proteasome pathway were both up-regulated in zebrafish liver and HepG2 cells. Likewise, nuclear receptor signaling pathway, mitochondrial fatty acid beta-oxidation and electron transport chain (8 h in zebrafish) were down-regulated by HgCl_2 _in both zebrafish liver and HepG2 cells, indicating similar mode-of-action of HgCl_2 _in these two models at certain levels (Tables 1 and 2).

**Table 2:** Gene Set Enrichment Analysis (GSEA) of transcriptome of mercury-treated human HepG2 cells.

Enriched processes	NES HepG2
Cell cycle	2.2425
Pyrimidine metabolism	2.049
DNA repair	1.6355
DNA damage signaling*	1.4957
Proteasome pathway*	1.5095
Electron transport chain*	-1.4248
Nuclear receptor signaling pathway*	-1.5093
Mitochondrial fatty acid beta-oxidation*	-1.7171

Biological pathways that are statistically enriched with false discovery rate (FDR) < 0.3 or nominal p-value (NP) < 0.1 are considered. * indicate pathways that are common in zebrafish and HepG2 cell treated with HgCl_2_.

However, unlike zebrafish, HepG2 cells showed up-regulation of cell cycle, pyrimidine metabolism and DNA repair owing to the proliferative state of HepG2 cells (Table 2). This may indicate differential toxicity responses between proliferative cell cultures and highly differentiated *in vivo *models. More importantly, being an *in vivo *model, the zebrafish liver data has an advantage to capture deregulated biological processes that involves interactions of various cell types and whole-organism physiological metabolic processes such as fatty acid synthesis, gluconeogenesis, and other processes involving non-substrate Gsk3 interacting proteins and complement activation. Thus, using an *in vivo *model such as zebrafish may help researchers to characterize pathways that are perturbed by toxicants under *in vivo *environments.

### HgCl_2 _affected liver cell morphology and cell adhesion

In order to further elucidate the mechanism of mercury-induced hepatotoxicity, we performed phenotypic anchoring [28,29] by linking histological evidence to the mercury-induced transcriptome changes. This was further validated by real-time PCR for targeted genes associated with mercury-induced phenotype/pathology and transcriptome changes in the liver. In these experiments, we treated a new batch of zebrafish with different concentrations of HgCl_2_, from environmental-relevant (10 and 50 μg/L) to sub-lethal concentration (200 μg/L). Real-time PCR validation of targeted genes were performed on liver samples from zebrafish treated with 10, 50 and 200 μg/L for 24 h while phenotype anchoring were performed using liver from zebrafish treated with 50 and/or 200 μg/L for 96 h.

**Figure 1:**
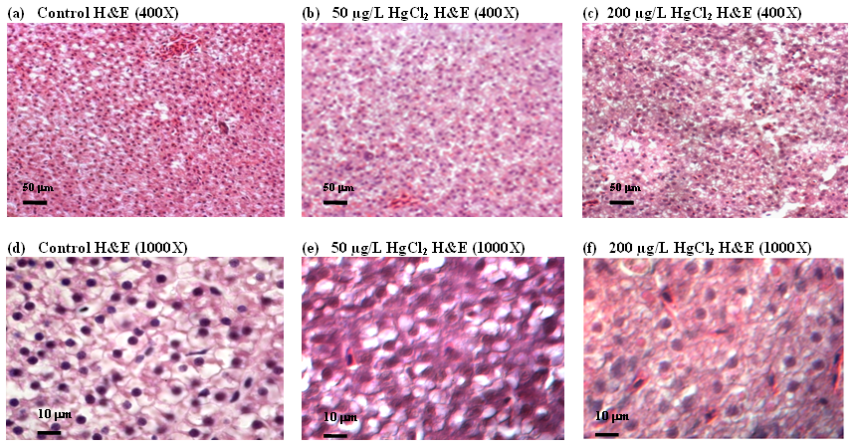
**Hematoxylin and eosin (H&E) staining of liver sections from zebrafish treated with mercury**. Adult zebrafish were treated with 0 (control, a and b), 50 mg/L (b and e) and 200 mg/L HgCl_2 _(c and f). Panels (a-c) are shown in low magnification (400×) while (d-f) in high magnification (1000×). HgCl_2_-treated livers show less compact, homogeneous distribution, more dissociated, irregular in shape and lack of delineated polygonal shape of hepatic parenchyma cells as compared to the controls.

Qualitative histological examinations for samples stained with hematoxylin and eosin (H&E) had revealed that hepatic parenchyma cells appeared to be less homogeneous in HgCl_2_-treated fish than in control fish (Figure 1). At a high magnification, it was observed that the liver parenchyma from HgCl_2_-treated fish appeared to be loose in cell contact and the cells were dissociated and irregular in shape (Figure 1b, 1c, 1e and 1f) while control fish liver is filled with well-delineated polygonal cells (Figure 1a and 1d).

**Figure 2:**
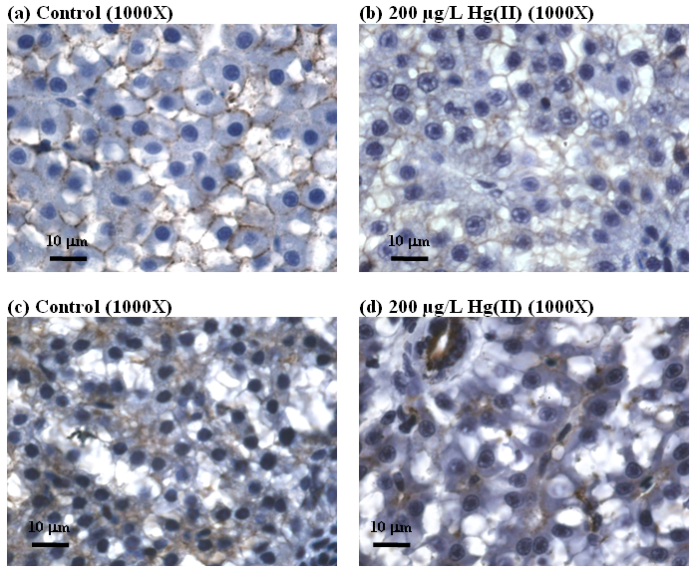
**Comparison of hepatic histopathological changes in cell-cell adhesion in control liver (a, c) and induced by HgCl_2 _(b and d)**. (a) and (b) show immunohistochemical staining for E. cadherin (indicated by dark brown precipitates). (c) and (d) show immunohistochemical staining for cytokeratin (indicated by dark brown precipitates).

Immunohistochemical staining of E-cadherin, a transmembrane cell adhesion protein, was weaker and less distinct between the liver cells in HgCl_2_-treated fish than control fish, suggesting that membrane integrity and cell adhesion were affected (Figure 2a and 2b). Likewise, immuno-staining of keratin-8 revealed a weaker staining of the cytoskeletal protein in liver cells of mercury-treated fish than those of control fish (Figure 2c and 2d), suggesting altered cytoskeletal organization and assembly which can affect cell morphology and motility. Thus, the histological phenotypes such as changes in cell morphology, cell adhesion and cytoskeletal proteins observed in the liver of HgCl_2_-treated zebrafish corroborated with the deregulated biological processes such as the actin pathway, Wnt signaling pathway and cell motility as identified in the transcriptome analysis.

### HgCl_2 _decreased hepatocyte cell number via induction of apoptosis

**Figure 3:**
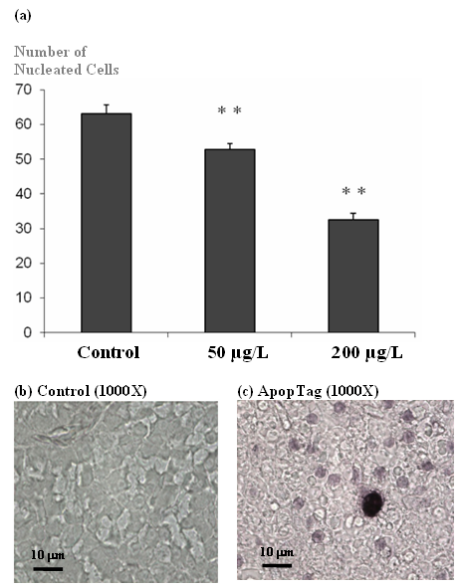
**Liver damage induced by HgCl_2_**. (a) Quantitative histological examinations of nucleated-hepatocyte cell count based on H&E stained sections for HgCl_2 _treated fish was observed to significantly decrease in concentration-dependent manner (** p value < 0.05) compared to the controls. (b) and (c) Apoptag staining for apoptosis-induced DNA damage in hepatocytes in control (b) and HgCl.-treated zebrafish liver (c).

Quantitative counting of nucleated hepatic cell counts based on H&E stained sections revealed that HgCl_2 _decreased the number of hepatic parenchyma cells significantly in a concentration-dependent manner (Figure 3a). The number of hepatic parenchyma cell decreased approximately 20% and 50% in livers of fish treated with 50 and 200 μg/L HgCl_2_, respectively, when compared to livers of control fish. Immunohistochemical-staining of DNA breakage induced by apoptosis detected higher incidence of apoptotic DNA fragments in livers of HgCl_2_-treated fish than control fish (Figure 3b to 3c). Transcriptome analysis supported this observation, as indicated by the fact that intrinsic apoptotic pathway was induced at 96 h by HgCl_2 _(Table 1). In addition, it has been shown that HgCl_2 _induced apoptosis in human leukemia cells via a mitochondrial-dependent intrinsic apoptotic pathway [30]. Therefore, our findings implicate that the decrease in liver cells is caused by induction of apoptosis via the mitochondrial intrinsic apoptotic pathway.

### HgCl_2 _induced fatty liver condition

**Figure 4:**
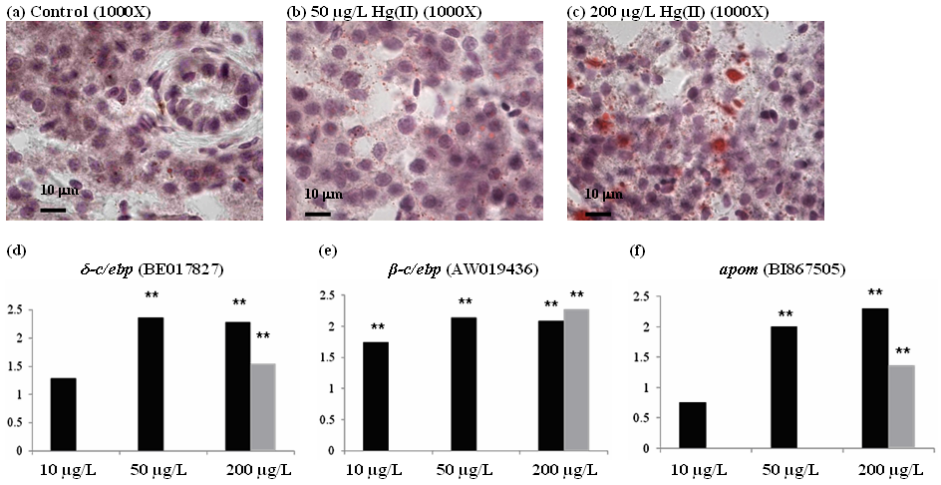
**Fatty acid accumulation in zebrafish liver upon mercury exporure**. (a-c) Oil red O staining for lipid content in control (a) and HgCl_2_-treated livers (b-c). Lipid content in the hepatocyte was stained and indicated by red staining showing increased of lipid content in liver of mercury-treated zebrafish is concentration-dependent (b-c). (e, f) Quantitative real-time PCR for mRNAs of adipogenesis genes *δ-c/ebp *(d), *β-c/ebp *(e) and *apom *(f). Results from Real-time PCR indicate level of induced gene expression in these genes are dependent to the concentration of HgCl_2 _and are sensitive to low concentration of HgCl_2 _at 10 μg/L. Light grey bar indicate data from microarray (** p-value < 0.05).

By oil red O staining, dosage-dependent lipid accumulation, as indicated by the increased number and size of red-stained lipid vesicles, was detected in the liver of HgCl_2_-treated fish (Figure 4a to 4c). The histological phenotype was thus consistent with the transcriptome analysis where up-regulation of fatty acid synthesis and down-regulation of mitochondrial fatty acid beta-oxidation were found in the liver of HgCl_2_-treated zebrafish (Table 1). Accumulation of lipids can lead to adipogenesis, steatostasis or non-alcoholic fatty liver diseases. The transcription factors CCAAT/enhancer-binding proteins (C/ebps) are known to modulate gene expression leading to adipogenesis [31,32]. Hence, we performed quantitative real-time PCR on *β-c/ebp, δ-c/ebp*, *apolipoprotein M (apom) *and found that they were indeed up-regulated by HgCl_2 _(Figure 4d, 4e, 4f) as suggested by transcriptome data. As shown in Figure 4d and 4e, both *β-c/ebp *and *δ-c/ebp *were induced by HgCl_2 _at concentrations as low as 10 μg/L, suggesting that these transcription factors were very sensitive to mercury-induced lipid accumulation in the liver and are potential biomarkers for mercury exposure as well as for fatty-liver condition.

### HgCl_2 _induced glycogen accumulation in liver

**Figure 5:**
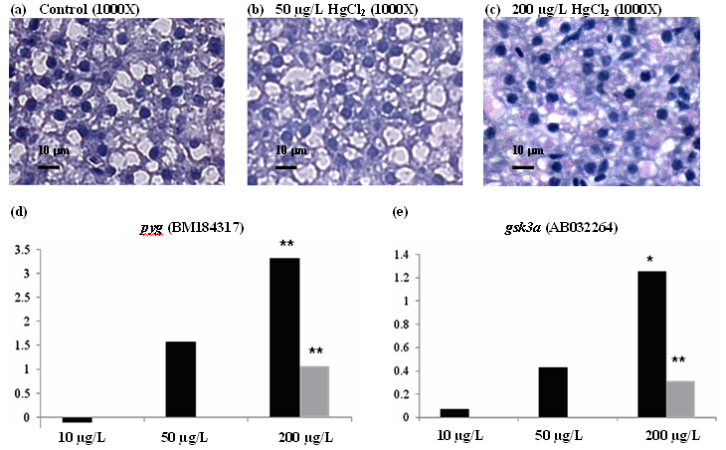
**Glycogen accumulation in zebrafish upon mercury exposure**. (a-c) Periodic acid Schiff staining (PAS) for glycogen content in control (a) and HgCl_2_-treated livers (b-c). Glycogen content in the hepatocyte was stained and indicated by pink staining. The increased of glycogen content in the liver of mercury-treated fish is concentration-dependent. (d and e) Quantitative real-time PCR for glycogen phosphorylase (*pyg*) and glycogen synthase kinase 3a (*gsk3a*) that involve in gluconeogenesis, respectively. Results from real-time PCR indicate induced gene expression in these genes are dependent to concentration of HgCl_2_. Light grey bar indicate data from microarray (* p-value < 0.1; ** p-value < 0.05).

By Periodic Acid-Schiff (PAS) staining, we observed increasing glycogen content, as indicated by the increased 'pink staining', in livers of zebrafish treated with increasing concentrations of HgCl_2 _(Figure 5a to 5c). The increase of glycogen content in liver of mercury-treated zebrafish correlated with the up-regulation of gluconeogenesis based on transcriptome data (Table 1). Real-time PCR confirmed that gene expression for both *glycogen phosphorylase (gp) *and *glycogen synthase kinase 3 (gsk3) *were up-regulated by HgCl_2 _in a concentration-dependent manner (Figure 5d and 5e). In addition, a previous study [33] has shown significant elevation of glycogen level in *vas deferens *of male rat treated with HgCl_2_, suggesting that mercury may cause metabolic disorders via increased level of glycogen.

### Comparative analysis revealed similarity of mercury- and arsenic-induced processes in zebrafish liver

We have previously reported that arsenic exposure in adult zebrafish caused decreased number of hepatic parenchyma cells as well as altered cell adhesion and cytoskeleton [23]. Due to similarity of the toxicity effects between these two compounds, we compared transcriptome profiles between mercury and arsenic in the zebrafish liver using GSEA. Similar statistical criteria were used to select enriched pathways induced by arsenic (See Additional file 3). Arsenic induced proteasome pathway and intrinsic apoptotic pathway as early as 8 h of arsenic exposure. Unlike mercury, extrinsic apoptotic pathway is not enriched. Also, DNA damage signaling pathway was significantly up-regulated at 96 h of arsenic treatment although positive NES values were observed at an earlier time point. Like the case of mercury, Wnt signaling was also significantly down-regulated in Asenic-treated livers. However, unlike mercury, gluconeogenesis and fatty acid synthesis were down-regulated by arsenic (See Additional file 3) implying that there are different toxicity modes between these two compounds.

### Validation of targeted genes as potential biomarkers of mercury-induced hepatotoxicity

**Figure 6:**
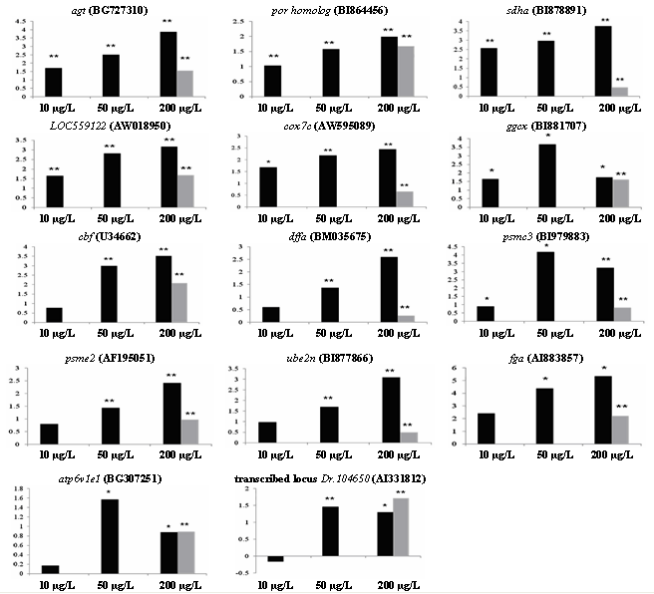
**Quantitative real-time PCR validation for selected genes**. The expression of these selected genes were based on stringent statistical criterion (FDR<0.0005) and/or biological function of interest. Most of these genes show concentration-dependent induction by HgCl_2 _that are also sensitive to low concentration at 10 μg/L, indicating the potential of these genes as markers for mercury-induced hepatotoxicity. Light grey bar indicate data from microarray (* p-value < 0.1; ** p-value < 0.05).

It is also important to identify genes whose expressions are sensitively induced by mercury exposure. Based on stringent statistical criterion (FDR<0.0005) and/or biological function of interest, we validated selected genes using real-time PCR. Our data indicated that 14 genes i.e. *fibrinogen alpha chain *(*fga*), *complement factor B *(*cfb*), *angiotensinogen *(*agt*), *P450 oxidoreductase *homolog (*por *homolog), *gamma-glutamyl carboxylase *(*ggcx*), hypothetical *LOC559122 *(which has predicted domain of complement control proteins), ubiquitin conjugation enzyme E2N *(ube2n)*, DNA fragmentation factor *(dffa)*, proteosome activator subunit 2 *(psme2)*, proteosome 26S subunit *(psmc3)*, cytochrome c oxidase subunit VIIC *(cox7c) *and succinate dehydrogenase complex subunit A *(sdha) *were up-regulated within 24 h of exposure to 200 μg/L of Hg(II) (Figure 6). This further confirmed biological processes such as proteasome degradation (*ube2n, psme2, psmc3*) and apoptosis *(dffa*), complement activation (*cfb*, hypothetical LOC559122) and electron transport chain (*por *homolog, *cox7c*, *sdha*), acute phase response (*fga*, *cfb, agt*) were deregulated. However, only 7 genes i.e. *por *homolog, *cox7c*, *sdha *(electron transport chain), hypothetical LOC559122, (predicted to associate with complement proteins) and *agt *(acute phase protein), and *β-c/ebp *and *δ-c/ebp *(for fatty liver condition, Figure 4d and 4e) were up-regulated by 10 to 200 μg/L of HgCl_2_, suggesting that oxidative phosphorylation, complement system, acute phase response and lipid metabolism in the liver are highly sensitive to mercury exposure. These findings further imply the susceptibility of the liver to oxidative stress, complement activation, acute phase response and steatosis as a consequence of mercury exposure. The environmental limit of inorganic mercuric from the United States Environmental Protection Agency (US EPA) in drinking water is below 2 ppb (2 μg/L) http://www.epa.gov/ogwdw000/contaminants/dw_contamfs/mercury.html. Mercury content in human blood is normally within the range of 0.87 to 3.51 μg/L [34,35] and the warning level of mercury in human blood is 22.8 to 30 μg/L [34,36]. The reproducible, rapid and dose-dependent responsiveness of these genes to a range of HgCl_2 _concentrations from 10 to 200 μg/L suggested that these genes can be potentially used as robust biomarkers in combination with analytical chemistry detection of mercury for investigating and assessing the severity of mercury exposure. Furthermore, related acute phase proteins such as angiotensinogen and complement factors that are synthesized in liver and secreted into blood and excreted in urine are potential clinical as well as toxicogenomic biomarkers for assessing health effects of mercury exposure and warrants further investigation [37].

### Mechanistic insights into *in vivo *mercury-induced hepatotoxicity

**Figure 7:**
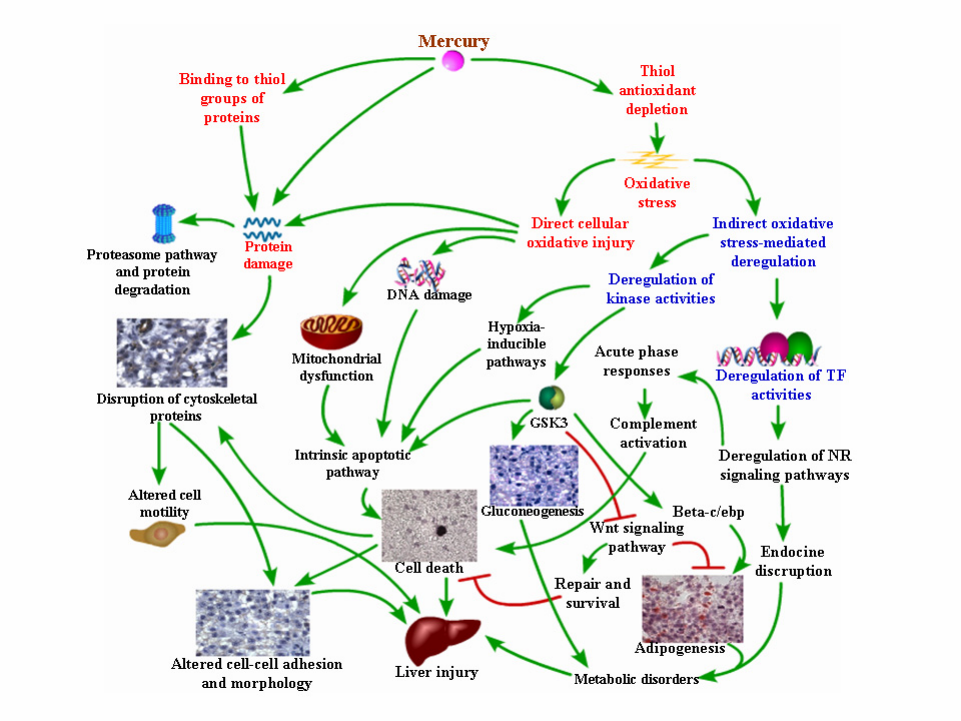
**Proposed *in vivo *hepatotoxicity mechanism in zebrafish upon mercury exposure**. The model is proposed based on our transcriptome analysis, phenotype anchoring and targeted gene validation. Words in red and blue fonts are direct and indirect upstream processes induced by mercury, respectively. Black words are downstream processes that are induced or suppressed by either direct or indirect effects of mercury. Please see text for detail description of the indicated mechanism.

Taken together, our findings provide the following *in vivo *mechanistic insights into mercury-induced hepatotoxicity (Figure 7). As a strong thiol-binding agent, mercury causes protein damage and depletion of thiol-containing antioxidants (red fonts in Figure 7). Protein damage subsequently induces proteasome pathways as well as further disruption of cellular structure such as cell-cell adhesion and cell motility. This may explain the very steep acute toxicity curve (See Additional file 1) due to the rapid increase of toxicity when protective intracellular thiol-antioxidants have been depleted after a threshold (~200 μg/L) of HgCl_2_. Transcriptome data showed deregulation of key genes in glutathione metabolism (*gstp, gstt1, pgd*), providing further evidence of disrupted thiol-antioxidants intracellular supply (See Additional file 4).

Depletion of thiol-containing antioxidants will cause direct (red fonts in Figure 7) and indirect (blue fonts in Figure 7) oxidative stress-mediated liver injury. Direct oxidative stress induces DNA damage, mitochondrial dysfunction such as increased oxidative phosphorylation or electron transport chain activity and reduced activity of fatty acid β-oxidation. These will subsequently cause induction of mitochondria-mediated intrinsic apoptotic pathway to release Caspase-8 to trigger apoptosis and cell death leading to liver injury. Depletion of thiol-antioxidants results in the overall intracellular redox balance to shift towards increased oxidative stress, especially in the mitochondria where active oxidative phosphorylation occurs. This is evident from the transcriptome analysis indicating the deregulation of electron transport chain and mitochondrial fatty acid beta-oxidation in at least 3 time-points (8 h, 24 h and 48 h; Table 1), suggesting mitochondrial dysfunction occurring as early as 8 h of mercury exposure. Moreover a shift from down-regulation of extrinsic apoptosis at 8 h time-point to up-regulation of mitochondrial-mediated intrinsic apoptosis signaling at 96 h time-point (Table 1) strongly suggests increased mitochondrial dysfunction and intracellular stress [38,39]. Increased apoptosis was confirmed through real-time PCR of up-regulated *dffa *(encoding a substrate of Caspase 3 that triggers DNA fragmentation during apoptosis) as showed in Figure 6 and immunohistochemical-staining of apoptotic DNA fragments in Figure 3. Some of the observed histological phenotypes in the liver parenchyma, such as irregular cell-shape, loose contact with neighboring cells and significantly reduced nucleated-cell count, may be attributed to morphological features of apoptosis [40] as well as to mercury interaction with cytoskeletal proteins [14]. E-cadherin and Keratin-8 staining further revealed the extent of the disrupted cell adhesion and cytoskeletal organization corroborating with the deregulated actin pathway and cell motility process captured by the transcriptome data. Unmitigated oxidative stress would cause cellular damage involving DNA and proteins and lead to tissue injury as well as cell lysis/death, as suggested by the up-regulation of DNA damage signaling, proteasome pathway, several acute phase protein genes (*fga*, *agt*) including complement activation. This further explains the down-regulation of cell cycle indicated in the transcriptome analysis which is necessary for DNA repair and assessment of cellular damage, and if necessary, to induce cell death instead of cell proliferation.

In addition to direct mercury-induced oxidative cellular injury, our study has also captured indirect mercury-induced oxidative stress-mediated gene expression/regulation (blue fonts in Figure 7). Depletion of cellular thiols and intracellular redox balance can alter activity in many transcription regulators and nuclear receptors [41,42] as indicated by the early deregulation nuclear receptor signaling pathway (Table 1) that include genes such as *esr1 *(*estrogen receptor 1*) and *nr2f1l*, further confirming the endocrine-disrupting effect of mercury [43]. Our findings suggest that the liver is a mercury-targeted organ for endocrine disruption. In addition to hormonal receptors, the up-regulation of transcription regulators such as *junb, β-c/ebp*, *δ-c/ebp*, and *keap1 *suggested modulation of protective/defense and survival mechanisms involving genes associated with antioxidant response element (ARE) (*keap1, junb, junc, gstp1, gclm, sqstm1*) and acute phase response (*β-c/ebp*, *δ-c/ebp, cfb, c3, c9, fga, fgb, hpx*) (See Additional file 4). Up-regulated glucocorticoid receptor signaling together with *β-c/ebp *could lead to gluconeogenesis and glycogen synthesis as indicated in the transcriptome analysis and PAS staining of glycogen as shown in Figure 5. Moreover, up-regulation *β-c/ebp *and *δ-c/ebp *showed in Figure 4 together with the disruption of mitochondrial fatty acid beta-oxidation could lead to fat synthesis and accumulation. All these incidents can eventually lead to metabolic disorders that may cause further liver injury.

Apart from transcription regulators, it was also observed that expression of genes encoding several kinases (*cdc2, gsk3a, mapkapk2, ptk2 *and *sgk*) were also affected suggesting that signal transduction via phosphorylation was altered by mercury-induce oxidative stress. Modulation of kinases involved in MAPK (Mitogen-Activated Protein Kinase) pathways could result in up-regulation of hypoxia signaling [44]. Furthermore, deregulated kinases such as Gsk3 will trigger hypoxia-inducible pathways and intrinsic apoptotic pathway. Gsk3 had been shown to activate intrinsic but not extrinsic apoptotic pathway [45] and deregulate Wnt signaling pathway [46,47] as indicated in the transcriptome analysis. Likewise, Wnt signaling can act as negative regulator for adipogenesis [31,32] as its down-regulation may also contribute to adipogenesis observed in the study.

Finally, it should be pointed out that the analysis is based on the nominal concentration of mercury used in the exposure experiment and does not reflect the amount of mercury bioaccumulated in the tissue. Future studies should include bioaccumulation of mercury in target tissues in order to relate the observed effects to its actual concentration in the tissues. This is important for assessing and comparing the actual degree of mercury exposure in the tissue with its corresponding toxicological responses from different studies.

## Conclusion

Our study revealed *in vivo *mechanistic insights of mercury-induced hepatotoxicity that may imply complications associated with metabolic disorders. To our knowledge, this is the first report of an *in vivo *mechanistic model for mercury-induced toxicity in the liver. Comparative analyses from arsenic-induced hepatotoxicity in zebrafish and human HepG2 cell line treated with HgCl_2 _showed some similar effects with mercury-induced hepatotoxicity in zebrafish. In addition, transcriptome analysis coupled with phenotypic anchoring and targeted gene validation had revealed that mercury can cause liver injury via broad processes associated to oxidative stress and cell death as well as deregulation of kinases such as *Gsk3 *in regulating the activities of *Wnt *signaling pathway, gluconeogenesis and adipogenesis that may eventually lead to syndromes such as mitochondrial dysfunction, endocrine disruption and metabolic disorders. Moreover, targeted gene validation also identified genes whose expressions are sensitive to mercury exposure suggesting their potential use for detecting or diagnosing mercury-induced toxicity. Thus, the understanding of the *in vivo *mechanism of hepatotoxicity using zebrafish could facilitate researches in toxicology and toxicogenomics, in particular *in vivo *heavy metal toxicity. These findings provide strong evidence of the feasibility of using zebrafish to model mercury-induced hepatotoxicity in human for risk assessment of mercury.

## Methods

### The zebrafish

Adult zebrafish (6 months - 1 year old) were obtained from a local fish farm. The fishes were allowed to acclimatize in aquaria for several days before transferred into smaller containers for mercury exposure. For all three types of experiments (i.e. microarray, histology and PCR biomarker validation), zebrafish were exposed to mercury at different concentrations for 96 h at a density of one fish/200 ml at 27 ± 2°C. De-chlorinated water and chemical were renewed daily. All experimental with animals were conducted following the guidelines of Institutional Animal Care and Use Committee (IACUC) and the procedures were approved by IACUC.

### Mercury treatment

HgCl_2 _or mercury (II) chloride [99.5% A.C.S reagent; Sigma, Aldrich] was chosen in consideration of its solubility in water that greatly facilitates daily preparation of chemical stock. Concentrations for treatment were chosen based on pre-LC50 and LC50 results. Concentration used for treatment is from 0.01 - 0.2 mg/L. Prior to obtaining the transcriptomic profiles of zebrafish, the appropriate dosage of HgCl_2 _needed was determined from acute toxicity tests. Survival analyses for zebrafish treated with various concentrations of HgCl_2 _were performed. The survival rate of sub-lethal 200 μg/L of HgCl_2 _was 90% at day 7 was chosen for subsequent microarray and toxicological studies. Treatment for microarray analyses was carried out in 4 days, using one concentration (200 μg/L) of HgCl_2 _with liver RNA sampling at 8, 24, 48, and 96 hours, respectively. Treatment was conducted using triplicate groups of four zebrafishes.

### DNA Microarray Experiments

Total RNAs from triplicates (each replicate consisted of liver pooled from four fishes) at the respective sampling time-points were isolated by Trizol reagent from Gibco-BRL. Reference RNA was obtained by pooling equal amount of total RNA extracted from male and female wildtype fish. Reference RNA was co-hybridized with RNA samples either from treated or control fish on a glass array spotted with 16,416 zebrafish oligo probes (16,244 LEADS clusters and 172 copies of β*-actin *controls) [48]. Both reference and sample RNAs were reverse-transcribed and labeled differently, using fluorescent dyes Cy-3 and Cy-5, respectively. After hybridization at 42°C for 16 hours in hybridization chambers (Gene Machines), the microarray slides were washed in a series of washing solutions (2× SSC with 0.1% SDS; 1× SSC with 0.1% SDS; 0.2× SSC and 0.05× SSC; 30 seconds each), dried by low-speed centrifugation and scanned for fluorescence detection using the GenePix 4000B microarray scanner (Axon Instruments). Further detailed protocols for microarray experiment and data acquisition have been recently published by us [49,50].

### Microarray data processing and transcriptome analysis

The raw microarray data was normalized using Lowess method in the R package http://www.braju.com/R/. The data had been submitted to Gene Expression Omnibus (GEO) with accession number of GSE18861. One-way ANOVA was performed on the normalized data followed by Student's t-test to identified genes whose expressions are significantly altered by HgCl_2_. Gene set enrichment analysis (GSEA) [27] was performed to characterize pathways or processes that are affected by HgCl_2_. Fishes were retreated with HgCl_2 _and quantitative real-time PCR was used to validate genes whose expressions were significantly altered as well as some regulatory genes in relevant pathways affected by HgCl_2_.

### Analysis of Transcriptomics Profile Using Gene Set Enrichment Analysis (GSEA)

Gene Set Enrichment Analyses (GSEA) as described in detail by [27] was used to determine biological pathways perturbed by HgCl_2_. The zebrafish genes were mapped to human homologous genes as previously described [22]. The human homologs of zebrafish genes from the HgCl_2_-induced transcriptome profiles were ranked based on their p-values using Student t-test. The "GSEAPreranked" option of GSEA was used. The ranking metric used was log10 (1/P) where P is the p-value of a gene from microarray data. Up-regulated genes will carry positive values of log10 (1/P) whereas down-regulated genes will carry negative values of log10 (1/P). The genes were then ranked in descending order based on values of log10 (1/P). The ranking procedure was conducted for array at each time point of HgCl_2 _treatment. The ranked list of genes for each time-point treatment will then be compared to 639 pre-defined gene sets or signatures of canonical pathways. These pre-defined gene sets can be downloaded from the GSEA website.

An enrichment score (ES) that reflects the degree to which a pre-defined gene set is overrepresented at the top or bottom rank of the ranked whole transcriptome profile was calculated by walking down the ranked profile. The ES is the maximum deviation from zero encountered in the random walk corresponds to a weighted Kolmogorov-Smirnov-like statistic. The statistical significant (nominal p-value, NP) of the ES was estimated by using an empirical phenotype-based permutation test procedure. The phenotype labels were permuted and the ES of the gene set for the permuted data were recomputed that generate a null distribution for the ES. The empirical, nominal p-value of the observed ES was then calculated relative to this null distribution. The estimated significance level was adjusted with multiple hypothesis testing. The ES for each gene set was first normalized to the size of the set yielding a normalized enrichment score (NES) with the following relation:

NES = actual ES/mean (ESs against all permutations of the dataset)

The number of permutation used was 1000. The proportion of false positive was then controlled by calculating the false discovery rate (FDR) corresponding to each NES.

Pathways with false discovery rate (FDR) < 0.3 or nominal p-value (NP) < 0.1 were considered statistically significant. Positive and negative values of normalized enrichment scores (NES) indicated the activities of pathways that were up- and down-regulated, respectively.

### Biomarker Validation with Real Time Quantitative PCR

The quantitative Real-Time PCR reaction was performed from synthesized FirstStrand cDNA using LightCycler^® ^480 SYBR Green I Master kit according to the manufacturer's protocol (Roche). Six biological replicates were performed for all real-time PCR experiments. Analysis of the transcript levels were performed by using relative quantification between the PCR signal of the target transcript in a treatment group and that of an untreated control group after normalization with the transcript level of beta-actin. The detail information of primers used is given in Additional file 5.

### Histological Sectioning and Sample Preparation

We repeated the treatment of zebrafish with HgCl_2 _for histological analysis. Adult zebrafish were treated with different concentrations (50 μg/L, 200 μg/L) of HgCl_2 _[99.5% A.C.S reagent; Sigma-Aldrich] for 96 hours at a density of 1 fish/200 ml at 27 ± 2°C in a static condition. Control fish were kept in water under similar conditions, and a total of 4 fish were used for each group. Treated solution and water were changed daily. Fish were slit open ventrally from heart to the anus to expose the digestive organs. The whole gut (alimentary tract including the liver) were removed and fixed in Formalin solution 10%, Neutral Buffered (Sigma-Aldrich) for 2-3 days at room temperature. Fixed gut samples were washed several times with 70% ethanol, followed by dehydration in a grade series of ethanol (70% - 100%) before clearing in Histoclear and embedding in paraffin. The paraffin-embedded tissues were sectioned serially at 5 μm thickness. The sections were stained with hematoxylin and eosin (H&E) for qualitative and quantitative histological analysis.

### Periodic acid-Schiff (PAS) staining

PAS staining is mainly used for staining structures containing a high proportion of carbohydrate macromolecules (glycogen, glycoprotein, proteoglycans) typically found in connective t/s, mucus and basal lamina). Staining for glycogen was performed using Alcian Blue PAS stain kit without diastase according to the manufacturer's protocol (BioGenex).

### Oil red O staining

Staining for adipocytes (Lipid and fat globules) was performed using Cryostat Sectioning from fresh frozen liver samples. Staining procedure was performed using Oil Red O stain according to the manufacturer's protocol (Sigma-Aldrich).

### ApopTag staining

DNA fragmentations associated with ultrastructural changes in cellular morphology in apoptosis were detected using ApopTag^®^Plus Fluorescein *In Situ *Apoptosis Detection Kit according to the manufacturer's protocol (Chemicon). The 3'-OH ends of double-stranded or single-stranded DNA were labeled with the digoxigenin-nucleotide and then allowed to bind an anti-digoxigenin antibody (Anti-DIG) that is coujugated to alkaline phosphatase. For The DNA fragmentations localized in apoptotic bodies were detected enzymatically using 5-Bromo-4-chloro-3-indolyl phosphate (BCIP)/Nitroblue tetrazolium (NBT) substrate.

### Histopathological examination

Histopathological analysis was performed to obtain a tissue-level image of HgCl_2 _effects on zebrafish at 96 hours. This also serves to further understand the gene expression profiles resulted from the microarray analyses. Imaging of liver sections was carried out using a compound microscope, Axioskop 2, Zeiss^® ^equipped with an imaging system and were analyzed with a computer-assisted image analyzer program (Axiovision, Zeiss). Liver sections from treated fish were compared with control fish, both quantitatively (i.e. cell size and cell density) and qualitatively (i.e. changes observed in liver tissue) from hematoxylin and eosin-stained section. Density of nucleated cells (no. of nucleated cells/5,100 μm^2^) and the size of nucleus were determined with an image analyzer in both the HgCl_2_-treated group and control group. Three images from each portion (anterior, middle and posterior portions) of liver sections (1,000× magnification) for each liver from four control and four HgCl_2_-treated fish (n = 4 liver samples) were used to determine the density of nucleated cells and to measure the size of the nuclei for each image. A two-tailed *t*-test was performed to analyze the data for statistical significance (*P *< 0.05).

Images of the PAS stained sections (1,000× magnifications) were captured using Axioskop 2 for each liver from four control and four HgCl_2_-treated fishes. Comparison of the glycogen content in control and treated liver samples were performed. Images of the Oil-red O stained sections (1,000× magnifications) were also captured using Axioskop 2. Comparison of the adipocytes (lipid globules) stained with Oil-red O from each image of control and treated liver sections were performed.

## Authors' contributions

Conceived and designed the experiments: SHL CYU CLW ZG. Analyzed the data: CYU SHL ZG. Contributed reagents/materials/analysis tools: CYU SHL MMH CLW SK SM ZG. Wrote the paper: CYU SHL ZG. All authors read and approved the final manuscript.

## Supplementary Material

Additional file 1 **Survival curves in response to HgCl_2 _exposure**. General acute toxicity test was conducted to determine the appropriate concentrations of mercuric chloride (HgCl_2_) for DNA microarray experiments using groups of 4 fishes. Survival curves with different labels show percentages of fish survival during the course of 7 days treatment with HgCl_2 _at the concentration of 200 μg/L, 300 μg/L, 400 μg/L and 600 μg/L, respectively. Click here for file

Additional file 2 **Number of deregulated genes (p-value < 0.05) by mercury that mapped to human homologous genes**. Bar graph in jpg format. Click here for file

Additional file 3 **Gene Set Enrichment Analysis (GSEA) of liver transcriptome of arsenic-treated zebrafish**. Biological pathways that are statistically enriched with false discovery rate (FDR) < 0.3 or nominal p-value (NP) < 0.1 are shown. Values of normalized enrichment score (NES) indicate the activities of enriched pathways with positive and negative NES shows up- and down-regulation, respectively. Click here for file

Additional file 4 **List of significant genes from one-way ANOVA with FDR < 0.05**. Values are in log2 scale in relative to levels of reference RNA. Click here for file

Additional file 5 **Detailed description of PCR primers used in this work**. Table in doc format. Click here for file
