# Supplementary figures and images for: Mercury-induced hepatotoxicity in zebrafish: in vivo mechanistic insights from transcriptome analysis, phenotype anchoring and targeted gene expression validation
Source: BMC Genomics. 2010 Mar 30;11:212. doi: 10.1186/1471-2164-11-212 (PMC2862047; doi:10.1186/1471-2164-11-212)

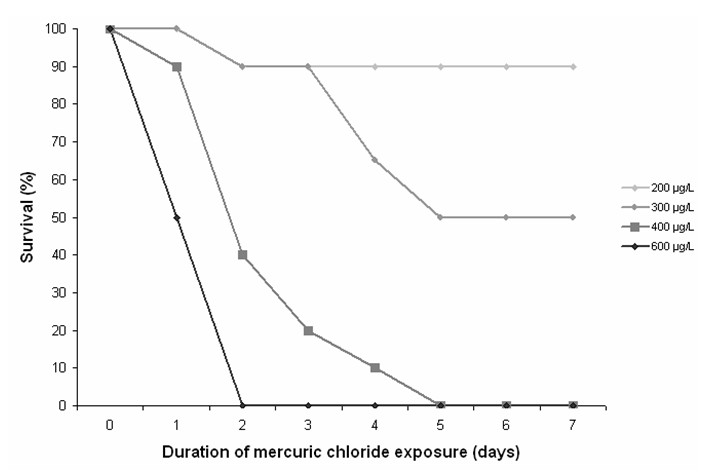

Supplement: Additional file 1 — Survival curves in response to HgCl2 exposure. General acute toxicity test was conducted to determine the appropriate concentrations of mercuric chloride (HgCl2) for DNA microarray experiments using groups of 4 fishes. Survival curves with different labels show percentages of fish survival during the course of 7 days treatment with HgCl2 at the concentration of 200 μg/L, 300 μg/L, 400 μg/L and 600 μg/L, respectively. [file 1471-2164-11-212-S1.JPEG]

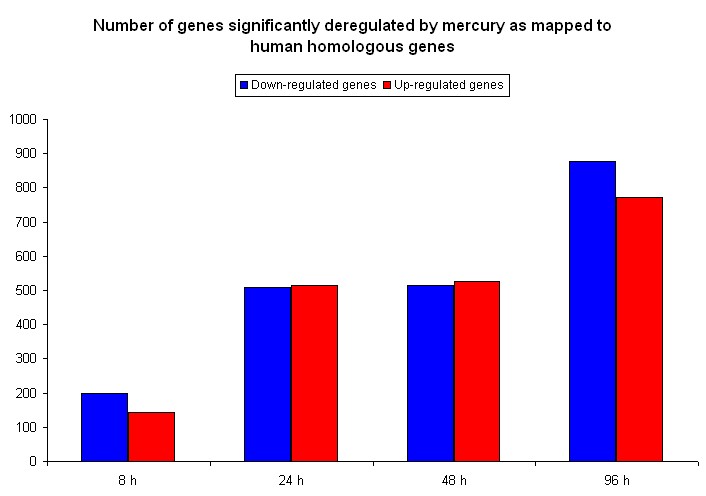

Supplement: Additional file 2 — Number of deregulated genes (p-value < 0.05) by mercury that mapped to human homologous genes. Bar graph in jpg format. [file 1471-2164-11-212-S2.JPEG]
