# Supplementary material for: Mercury-induced hepatotoxicity in zebrafish: in vivo mechanistic insights from transcriptome analysis, phenotype anchoring and targeted gene expression validation
Source: BMC Genomics. 2010 Mar 30;11:212. doi: 10.1186/1471-2164-11-212 (PMC2862047; doi:10.1186/1471-2164-11-212)
Supplement: Additional file 3 — Gene Set Enrichment Analysis (GSEA) of liver transcriptome of arsenic-treated zebrafish. Biological pathways that are statistically enriched with false discovery rate (FDR) < 0.3 or nominal p-value (NP) < 0.1 are shown. Values of normalized enrichment score (NES) indicate the activities of enriched pathways with positive and negative NES shows up- and down-regulation, respectively. [file 1471-2164-11-212-S3.DOC]

**Additional File 3**. Gene Set Enrichment Analysis (GSEA) of liver transcriptome of arsenic-treated zebrafish. Biological pathways that are statistically enriched with false discovery rate (FDR) < 0.3 or nominal p-value (NP) < 0.1 are shown. Values of normalized enrichment score (NES) indicate the activities of enriched pathways with positive and negative NES shows up- and down-regulation, respectively.

|  | **Up-regulated** | **NES** | **Down-regulated** | **NES** |
| --- | --- | --- | --- | --- |
| **8 h** | Cell cycle | 1.1628 | Complement activation (classical) | -1.3289 |
|  | Intrinsic apoptotic pathway | 1.2885 | Electron transport chain | -1.9019 |
|  | Proteasome pathway | 1.7097 | Fatty acid metabolism | -2.1169 |
|  |  |  | Mitochondrial fatty acid beta-oxidation | -2.0214 |
|  |  |  | Nuclear receptor signaling pathway | -1.2051 |
|  |  |  | Pyrimidine metabolism | -1.2547 |
|  |  |  | TCA cycle | -1.65 |
| **24 h** | Intrinsic apoptotic pathway | 1.443 | Cell motility | -1.141 |
|  | Proteasome pathway | 2.2261 | Circadian pathway | -1.4389 |
|  | Signaling proteins as substrates of GSK3 | 1.2773 | Complement activation (classical) | -1.3011 |
|  | Tumor necrosis factor pathway | 1.4811 | Fatty acid metabolism | -1.8083 |
|  |  |  | Gluconeogenesis | -1.4192 |
|  |  |  | Mitochondrial fatty acid beta-oxidation | -1.6271 |
|  |  |  | Negative regulators of GSK3 | -1.5134 |
|  |  |  | Non-substrate GSK3 interacting proteins | -1.4196 |
|  |  |  | Nuclear receptor signaling pathway | -1.9371 |
|  |  |  | Wnt signaling pathway | -1.2562 |
| **48 h** | Actin pathway | 1.2025 | Circadian pathway | -1.6223 |
|  | Electron transport chain | 1.1987 | Fatty acid metabolism | -1.7256 |
|  | Intrinsic apoptotic pathway | 1.3182 | Gluconeogenesis | -1.3281 |
|  | Proteasome pathway | 1.9403 | Mitochondrial fatty acid beta-oxidation | -1.8763 |
|  | Tumor necrosis factor pathway | 1.621 | Negative regulators of GSK3 | -1.1197 |
|  |  |  | Nuclear receptor signaling pathway | -1.9372 |
| **96 h** | Cytoskeleton | 1.3374 | Akt pathway | -1.1998 |
|  | DNA damage signaling pathway | 1.1931 | Fatty acid metabolism | -1.7967 |
|  | Electron transport chain | 1.2418 | Gluconeogenesis | -1.561 |
|  | Proteasome pathway | 2.103 | Mitochondrial fatty acid beta-oxidation | -1.8153 |
|  | Tumor necrosis factor pathway | 1.8064 | Negative regulators of GSK3 | -1.1234 |
|  |  |  | Nuclear receptor signaling pathway | -1.2813 |
|  |  |  | Pyrimidine metabolism | -1.5382 |
|  |  |  | Rho pathway | -1.2651 |
|  |  |  | Wnt signaling pathway | -1.11 |
