# Supplementary material for: Mercury-induced hepatotoxicity in zebrafish: in vivo mechanistic insights from transcriptome analysis, phenotype anchoring and targeted gene expression validation
Source: BMC Genomics. 2010 Mar 30;11:212. doi: 10.1186/1471-2164-11-212 (PMC2862047; doi:10.1186/1471-2164-11-212)
Supplement: Additional file 5 — Detailed description of PCR primers used in this work. Table in doc format. [file 1471-2164-11-212-S5.DOC]

**Additional File 5.**  Detailed description of PCR primers used in this work.

| **Gene Symbol** | **Gene Name** | **Gene ID** | **Product length** | **Annealing Temperature** | **Sense primer** | **Antisense Primer** |
| --- | --- | --- | --- | --- | --- | --- |
| *fga* | Fibrinogen alpha chain | AI883857 | 190-bp | 58ºC | CGGCTTGAGGTTGACATT | CATTCATCGGCNTGCTCT |
|  | Hypothetical LOC559122 | AW018950 | 142-bp | 58ºC | TGGAGTTGCCTTTCATACG | CCCTGTTCAGTGACATTGG |
| *cfa* | Complement factor B | U34662 | 164-bp | 58ºC | GCTTGTGGATGCTGCTTTC | TTTGTAACTGCCTCCGTCG |
|  | Transcribed locus | AI331812 | 117-bp | 58ºC | TCAAAGTAACCAACAAATGTCTAGG | CCTCATGATTTTGTTTTGCGTTAC |
| *agt* | Angiotensinogen | BG727310 | 171-bp | 58ºC | AGCTTGTCTAAACAAACATATCACC | ATCGCAGTTTTGGTGATGAC |
| *P450* | P450 oxidoreductase (Human homologue) | BI864456 | 188-bp | 58ºC | GCAATCGGAAACAACAGC | GTATGAGGGTGGACGACTTC |
|  | Gamma-glutamyl carboxylase | BI881707 | 170-bp | 58ºC | GAACTCAATTTAGGGGGAAAAAC | CCGGTTTGAAACAAGTAAAGG |
| *malt1* | Mucosa associated lymphoid tissue lymphoma translocation gene 1 | AF316598 | 245-bp | 62ºC | GCCTCTGAAACAACCTCAGC | CGCTGAAGGATGAAGAGTCC |
| *ube2n* | Ubiquitin-conjugating enzyme E2N | BI877866 | 257-bp | 57ºC | AAGCACGCAATGATGGAG | TCACCTGGGAAATGGTTC |
| *thy1* | Thy-1 cell surface antigen | BM181792 | 219-bp | 61ºC | CAGTGGGAAAGTGAGGAAGG | CGTGCCAATGTCACCATTAC- |
| *dffa* | DNA fragmentation factor, alpha polypeptide | BM035675 | 245-bp | 62ºC | ACAGAAGTGCTACGGACTGG | ACTCTCTACAAGACTGGGCTG |
| *psme2* | Proteasome activator subunit 2 | AF195051 | 267-bp | 62ºC | CAGAGGACGAGGAGATGGAG | CGACTTTGGTCTTCACAGCA |
| *psmc3* | Proteasome (prosome, macropain) 26S subunit, ATPase, 3 | BI979883 | 205-bp | 59ºC | CAAGTTTACAGGTGTGCTGG | TTTGTGTTGAGGCTGGTATG |
| *cox7c* | Cytochrome c oxidase, subunit VIIc | AW595089 | 222-bp | 60ºC | TTCGCTCCAGTCACTATGC | ATCCACACCAGAAGAAACAGAG |
| *sdha* | Succinate dehydrogenase complex, subunit A, flavoprotein (Fp) | BI878891 | 290-bp | 59ºC | GAGCAAATGTGATGGATGC | TCTGGACGCTGAAGACTGT |
| *atp6v1e1* | ATPase, H+ transporting, lysosomal, V1 subunit E isoform 1 | BG307251 | 229-bp | 63ºC | ACGCACACACACACACACAC | ACACTCTGGAGAGCCGACTG |
| *pyg* | Phosphorylase, glycogen | BM184317 | 241-bp | 58ºC | AGAACTTTCCCGATGCTG | TATGAGCGTCTGCCTGAA |
| *gsk3A* | Glycogen synthase kinase 3 alpha | AB032264 | 247-bp | 61ºC | TGGGAAAGTGACGACAGTAG | GAAGAAGTAGCGTAGCCTGAC |
| *cebpD* | CCAAT/enhancer binding protein (C/EBP), delta | BE017827 | 200bp | 60ºC | TTGGCAGGGTTTTGTTTTTC | TGCAGCAAAAGATGATCGAG |
| *cebpB* | CCAAT/enhancer binding protein (C/EBP), beta | AW019436 | 234bp | 60ºC | CGCACAGTCCACTTTCATGT | GAACGACGGGTTACAGAAGC |
| *apoM* | Apolipoprotein M | BI867505 | 169bp | 60ºC | CTGTATCGCCTCTTCTGTTG | GACAAGCCCTTTCCTGTTC |
